# Detection in two hospitals of transferable ceftazidime-avibactam resistance in *Klebsiella pneumoniae* due to a novel VEB β-lactamase variant with a Lys234Arg substitution, Greece, 2019

**DOI:** 10.2807/1560-7917.ES.2020.25.2.1900766

**Published:** 2020-01-16

**Authors:** E. Voulgari, S.D. Kotsakis, P. Giannopoulou, E. Perivolioti, L.S. Tzouvelekis, V. Miriagou

**Affiliations:** 1 Laboratory of Bacteriology, Hellenic Pasteur Institute, Athens, Greece; 2 Department of Clinical Microbiology, Thriassio General Hospital, Elefsina, Greece; 3 Department of Clinical Microbiology, Evangelismos General Hospital, Athens, Greece; 4 Department of Microbiology, Medical School, University of Athens, Athens, Greece

**Keywords:** Ceztazidime-avibactam, VEB β-lactamase, resistance, *Klebsiella pneumoniae*, pandrug, MDR, XDR

## Abstract

Two ceftazidime-avibactam (CAZ-AVI)-resistant *Klebsiella pneumoniae* carbapenemase (KPC)-positive *K. pneumoniae* strains, including one pandrug resistant, were isolated in 2019 from two Greek hospitals. The strains were sequence types (ST)s 258 and 147 and both harboured similar self-transmissible IncA/C2 plasmids encoding a novel Lys234Arg variant of the Vietnamese extended-spectrum β-lactamase (VEB)-1, not inhibited by AVI (VEB-25). Conjugal transfer of VEB-25-encoding plasmids to *Escherichia coli* yielded CAZ-AVI-resistant clones, supporting that VEB-25 is directly linked to the derived phenotype.

Ceftazidime-avibactam (CAZ-AVI) is a combination medication, which is active against various multidrug-resistant Gram-negative bacteria including *Klebsiella pneumoniae* carbapenemase (KPC)-*K. pneumoniae* (Kp) [1]. CAZ-AVI resistance among KPC-Kp strains is still rare and commonly associated with selection of KPC-2/3-derived mutant β-lactamases, resisting inhibition by avibactam [2-5]. In 2019, in view of the rapid risk assessment raising awareness on the emergence of resistance to ceftazidime-avibactam in carbapenem-resistant Enterobacteriaceae in Europe issued by the European Centre for Disease Control and Prevention (ECDC) [6], KPC-Kp isolates referred to Hellenic Pasteur Institute from Greek hospitals were further investigated. CAZ-AVI resistance was confirmed in two of 118 isolates analysed during 2019 and was attributed to the production of a novel Vietnamese extended-spectrum β-lactamase (VEB)-type. Herein, we describe the characteristics of the isolates possessing this resistance mechanism.

## Isolation of ceftazidime-avibactam-resistant strains and antibiotic susceptibility

The first CAZ-AVI resistant KPC-Kp strain (T-970/19) was isolated from blood cultures obtained from a female patient in her 60s hospitalised in the intensive care unit (ICU) of Hospital A. The patient was transferred in July 2019 from another hospital following a prolonged complicated hospitalisation course due to cardiopulmonary arrest and acute respiratory distress syndrome. On Day 10 of hospitalisation, a central line-associated bloodstream infection was identified whereupon blood cultures yielded an isolate (T-970/19) that exhibited an extensively drug-resistant phenotype retaining solely intermediate susceptibility to tigecycline. The second CAZ-AVI-resistant KPC-Kp isolate (E-1037/19) was retrieved from a male patient in his 30s admitted in August 2019 to the ICU of Hospital B with an epidural haematoma following a traffic accident. The E-1037/19 isolate was obtained on Day 14 of hospitalisation from a bronchoalveolar lavage fluid and exhibited resistance to all clinically available antimicrobials (pandrug resistant). Given the patient’s subsequent clinical course and laboratory findings this was considered as a colonisation. None of the patients had received CAZ-AVI before the isolation of the resistant strains.

## Whole genome sequencing and analysis

Genomic DNA was sequenced on a S5-Ion System platform. In silico multilocus sequence typing (MLST) and capsular polysaccharide (cps)-typing assigned T-970/19 to sequence type (ST)147 (*wzi*64) and E-1037/19 to ST258 clade I (*wzi*154). Resistance genes were identified using the nucleotide-nucleotide Basic Local Alignment Search Tool (blastn) programme of the BLAST + package and the gene sequences in the Bacterial Antimicrobial Resistance Reference Gene Database (https://www.ncbi.nlm.nih.gov/bioproject/PRJNA313047) of the National Center for Biotechnology Information (NCBI) as reference. The likely plasmid content was assessed with PlasmidFinder (http://cge.cbs.dtu.dk/services/PlasmidFinder/). T-970/19 and E-1037/19 contained *bla_K_*_PC-2_ and *bla*_KPC-3_, respectively. Both strains harboured *bla*_TEM-1_, *bla*_OXA-10_ and a *bla*_VEB_-type gene which, compared with *bla*_VEB-1_ (GenBank accession number: NG_050317) showed a non-synonymous mutation (A710G) leading to a novel Lys234Arg VEB mutant designated VEB-25 (GenBank accession number: MN862699). Notably, the Lys234Arg mutation has been observed in another VEB variant (VEB-20), which was reported in *Pseudomonas aeruginosa* from France (GenBank accession number: NG_063894), however the VEB-20 enzyme has an additional substitution compared to VEB-1 and VEB-25. Genes conferring resistance to aminoglycosides, fosfomycin, macrolides, phenicols, quinolones, rifampicin, sulfonamides, tetracycline and trimethoprim were also detected in both strains (Table 1). T-970/19 contained five replicons (IncA/C2, IncR, IncFIB(pKPHS1), IncFII(K), IncFIB(pQil)) while four replicons (IncA/C2, IncFIB(K), IncX3, ColRNAI) were identified in E-1037/19.

**Table 1:** Antibiotic resistance genes of Vietnamese extended-spectrum β-lactamase (VEB)-producing *Klebsiella pneumoniae* clinical strains and *Escherichia coli* transconjugant clones

Characteristics	T-970/19	TrcT-970	E-1037/19	TrcE-1037	TrcS-2865
**Affected antibiotic class**	**Resistance genes per isolate**				
Aminoglycosides	*aadA1*	*aadA1*	*aadA1*	*aadA1*	*aadA1*
	*aadA2*	*aadA2*	*aadA2*	NP	NP
	*ant(2”)-Ia*	*ant(2”)-Ia*	*ant(2”)-Ia*	*ant(2”)-Ia*	*ant(2”)-Ia*
	*aph(3”)-Ia*	*aph(3”)-Ia*	*aph(3”)-Ia*	NP	NP
	*aph(3”)-Ib*	*aph(3”)-Ib*	*aph(3”)-Ib*	*aph(3”)-Ib*	*aph(3”)-Ib*
	*aph(6)-Id*	*aph(6)-Id*	*aph(6)-Id*	*aph(6)-Id*	*aph(6)-Id*
	*rmtB1*	*rmtB1*	*rmtB1*	*rmtB1*	*rmtB1*
	NP	NP	*aac(6')-Ib-cr*	NP	NP
β-lactams	*bla*_KPC-2_	NP	*bla*_KPC-3_	NP	NP
	*bla*_VEB-25_	*bla*_VEB-25_	*bla*_VEB-25_	*bla*_VEB-25_	*bla*_VEB-1_
	*bla*_OXA-10_	*bla*_OXA-10_	*bla*_OXA-10_	*bla*_OXA-10_	*bla*_OXA-10_
	*bla*_TEM-1_	*bla*_TEM-1_	*bla*_TEM-1_	*bla*_TEM-1_	*bla*_TEM-1_
	*bla*_SHV-11_	NP	*bla*_SHV-182_	NP	NP
Fluoroquinolones (PMQR)	*oqxA*	NP	*oqxA*	NP	NP
	*oqxB*	NP	*oqxB*	NP	NP
Fosfomycin	*fosA*	*fosA*	*fosA*	NP	NP
Macrolides	*mdf*(A)	*mdf*(A)	*mdf*(A)	*mdf*(A)	*mdf*(A)
	NP	NP	*mph*(A)	NP	NP
Phenicol	*cmlA1*	*cmlA1*	*cmlA1*	*cmlA1*	*cmlA1*
	*floR2*	*floR2*	*floR2*	*floR2*	*floR*
	NP	NP	*catA1*	NP	NP
Rifampicin	*arr-2*	*arr-2*	*arr-2*	*arr-2*	*arr-2*
Sulfonamide	*sul1*	*sul1*	*sul1*	*sul1*	*sul1*
	*sul2*	*sul2*	*sul2*	*sul2*	*sul2*
Tetracycline	*tet*(A)	*tet(*A)	*tet*(A)	*tet*(A)	*tet*(A)
	*tet*(G)	*tet*(G)	*tet*(G)	*tet*(G)	*tet*(G)
Trimethoprim	*dfrA12*	*dfrA12*	*dfrA12*	NP	NP
	NP	NP	*dfrA14*	*dfrA14*	*dfrA14*
	NP	NP	*dfrA23*	*dfrA23*	*dfrA23*
**Plasmid replicon types**	**Plasmid replicon types per isolate**				
	IncA/C2 IncR, IncFIB(pKPHS1), IncFIB(pQil), IncFII(K)	IncA/C2 IncR	IncA/C2	IncA/C2	IncA/C2
	IncR	IncR	NP	NP	NP
	IncFIB(pKPHS1)	NP	IncFIB(K)	NP	NP
	IncFIB(pQil)	NP	ColRNAI	NP	NP
	IncFII(K)	NP	IncX3	NP	NP

NP: Not present; KPC: *Klebsiella pneumoniae* carbapenemase; OXA: oxacillinase; PMQR: plasmid-mediated quinolone resistance; SHV: sulfhydryl reagent variable β-lactamase; TEM: Temoniera β-lactamase; Trc: transconjugant; VEB: Vietnamese extended-spectrum β-lactamase.

T-970/19 and E-1037/19 are the VEB-producing *Klebsiella pneumoniae* clinical strains detected in this study. TrcT-970 and TrcE-1037 are the *Escherichia coli* transconjugant clones obtained by mating experiments using T-970/19 and E-1037/19 as donors and a β-lactam-susceptible *E. coli* K12 strain (26R793) as recipient.TrcS-2865 is a ceftazidime-avibactam-susceptible *E. coli* 26R793 transconjugant containing *bla*_VEB-1_, *bla*_OXA-10_ and *bla*_TEM-1_ that was used for control purposes.

## Transferability of ceftazidime-avibactam resistance and the role of the Vietnamese extended-spectrum β-lactamase-25

Mating experiments using T-970/19 and E-1037/19 as donors and a β-lactam-susceptible *Escherichia coli* K12 strain (26R793) as recipient yielded CAZ-AVI-resistant transconjugants (TrcT-970, TrcE-1037) at a frequency of 5×10 ^− 6^ per donor cell. Carriage of *bla*_VEB-25_, *bla*_OXA-10_ and *bla*_TEM-1_ by the transconjugant clones was confirmed by sequencing of PCR products. For control purposes, a CAZ-AVI-susceptible *E. coli* 26R793 transconjugant (TrcS-2865) containing *bla*_VEB-1_, *bla*_OXA-10_ and *bla*_TEM-1_ was used. Production of the respective β-lactamases was confirmed by isoelectric focusing of bacterial cell extracts. The apparent isoelectric point of VEB-25 was 7.5.

TrcT-970 and TrcE-1037 were resistant to piperacillin-tazobactam, ceftazidime, ceftazidime-avibactam, cefotaxime and aztreonam yet remained susceptible to carbapenems according to the European Committee on Antimicrobial Susceptibility Testing (EUCAST) interpretation criteria [7]. The VEB-1-producing TrcS-2865 displayed resistance to ceftazidime, cefotaxime and aztreonam (Table 2). Since OXA-10 and TEM-1 do not hydrolyze ceftazidime while VEB-1 is a potent ceftazidimase [8], comparison of susceptibility data indicated that VEB-25 (i) inactivated ceftazidime and (ii) was not inhibited by avibactam. To test the latter hypothesis, the ability of avibactam to inhibit β-lactam hydrolysis by VEB-25 and VEB-1 was comparatively assessed. Experiments were carried out using cell extracts of TrcE-1037 and TrcS-2865 as sources of VEB enzymes and ceftazidime as a substrate. Inhibition caused by avibactam was measured by spectrophotometry as described previously [9]. Results, expressed as the concentration (nM) of inhibitor required to reduce hydrolysis rates by 50% (IC50), showed that VEB-25 preparations were considerably less inhibited by avibactam (IC50 > 1,000 nM) as compared with those containing VEB-1 (IC50 = 0.1 nM).

**Table 2:** β-lactamase content and susceptibility to selected β-lactam antibiotics of Vietnamese extended-spectrum β-lactamase (VEB)-producing *Klebsiella pneumoniae* clinical strains and *Escherichia coli* transconjugant clones

Isolates	β-lactamase content	Etest MICs (μg/mL)						
		Ceftazidime	Ceftazidime -avibactam	Cefotaxime	Aztreonam	Piperacillin- tazobactam	Imipenem	Meropenem
T-970/19	KPC-2, VEB-25, TEM-1, OXA-10, SHV-182	> 256	64	32	> 256	> 256	> 32	> 32
TrcT-970	VEB-25, TEM-1, OXA-10,	> 256	32	4	> 256	16	0.25	0.032
E-1037/19	KPC-3, VEB-25, TEM-1, OXA-10, SHV-11	> 256	> 256	128	> 256	> 256	> 32	> 32
TrcE-1037	VEB-25, TEM-1, OXA-10,	> 256	128	12	> 256	24	0.25	0.023
ΤrcS-2865	VEB-1, TEM-1, OXA-10	> 256	0.25	4	128	2	0.19	0.023
26R793	None	0.5	0.094	0.047	0.125	1	0.19	0.016

KPC: *Klebsiella pneumoniae* carbapenemase; MIC: minimum inhibitory concentration; OXA: oxacillinase; SHV: sulfhydryl reagent variable β-lactamase; TEM: Temoniera β-lactamase; Trc: transconjugant; VEB: Vietnamese extended-spectrum β-lactamase.

T-970/19 and E-1037/19 are the VEB-producing *Klebsiella pneumoniae* clinical strains detected in this study. TrcT-970 and TrcE-1037 are the *Escherichia coli* transconjugant clones obtained by mating experiments using T-970/19 and E-1037/19 as donors and a β-lactam-susceptible *E. coli* K12 strain (26R793) as recipient.TrcS-2865 is a ceftazidime-avibactam-susceptible *E. coli* 26R793 transconjugant containing *bla*_VEB-1_, *bla*_OXA-10_ and *bla*_TEM-1_ that was used for control purposes.

## Structure of the Vietnamese extended-spectrum β-lactamase-25-encoding plasmids

The sequences of *bla*_VEB-25_-carrying plasmids were determined after whole genome sequencing (WGS) of transconjugants TrcT-970 and TrcE-1037 following the bioinformatic subtraction of the DNA sequence of *E. coli* 26R793 (the complete sequences of Trc970-T and Trc1037-E have been deposited in GenBank under the accession numbers WUBH00000000 and WUBG00000000). The size of pT-970 and pE-1037 plasmids were ca 170 and 150 kbp, respectively. Both plasmid sequence’s scaffolds exhibited high similarity scores (> 99%) with previously published IncA/C2 plasmids (GenBank accession number: CP027055) including the repA region, an intact conjugal transfer region and a parAB partitioning system. In pT-970 plasmid an additional IncR-derived segment including the repB replicase, a partitioning system and the vagCD and umuCD operons were identified. pT-970 and pE-1037 were punctuated by intact or truncated insertion sequences (IS) such as IS*26*, IS*6100*, IS*1999*, IS*1*, IS*3*-like, IS*L3* and IS*CR6*. Both plasmids contained the same multiresistant region (*Δ*IS*26* - IS*1999* - *bla*_VEB-25_ - *aadB* - *arr-2* - *cmLA5* - *bla*_OXA-10_ - *aadA1* - *qacEdelta1 -* *Δsul1* - *floR2 - tetR - tet*(G) *- lysR* - IS*CR3*-like - *groEL* - IS*L3* *- rmtB1* - *bla*_TEM-1_).

## Discussion

The presented findings clearly indicate that production of VEB-25 was responsible for resistance to CAZ-AVI. The enzyme was resistant to inhibition by AVI compared with its parental extended spectrum β-lactamase (ESBL) VEB-1. Moreover, it appears that VEB-25 retained the extended-spectrum properties of the VEB lineage. Similarly to VEB-25, substitution of the conserved lysine at Ambler’s position 234 by arginine in laboratory and natural variants of SHV and KPC class A β-lactamases confers resistance to inactivation by AVI and mechanism-based inhibitors [10-14]. It has been hypothesised that replacement of Lys234 by the longer side-chain Arg may alter the interaction with the adjacent Ser130 and consequently affect the catalytic process [10,14]. Yet, the mechanism remains unclear.

VEB-type β-lactamases are almost invariably associated with IncA/C2 replicons worldwide [15]. Enterobacteriaceae, mostly *K. pneumoniae* and *Providencia stuartii*, harbouring VEB-encoding multiresistant IncA/C2 plasmids have long been established in Greek hospitals occasionally causing outbreaks [16,17]. Indeed, during the course of this study 10% of the KPC-Kp were *bla*_VEB_-positive. Although the VEB-25 producers may have been selected by prior CAZ-AVI use in the population, they were acquired during hospitalisation in two patients who had not received the drug. It is therefore reasonable to assume that these strains were already part of the nosocomial flora. Our sampling approach unfortunately, is unable to accurately estimate the extent of dissemination of VEB-25 producers within the Greek Kp hospital populations. This is primarily due to the fact that the collection under investigation comprised only carbapenem-resistant isolates from either confirmed or suspected infections while neither carbapenem-susceptible nor surveillance culture isolates were included. Furthermore, participating hospitals are located solely in the Attika region. Whatever the case may be, the use of CAZ-AVI (introduced in this country in December 2017) will probably increase the clinical relevance of VEB-25-mediated resistance, given that the respective gene is carried by self-transferable plasmids that have been already acquired by high risk Kp clones potentially resulting in pandrug resistance.
